# Supplementary material for: Thermal and nutrient stress drove Permian–Triassic shallow marine extinctions
Source: Camb Prism Extinct. 2024 May 13;2:e9. doi: 10.1017/ext.2024.9 (PMC11895742; doi:10.1017/ext.2024.9)
Supplement: Foster et al. supplementary material [file S2755095824000093sup001.docx]

Supplementary Materials for

**Thermal and nutrient stress drove Permian-Triassic marine extinctions**

William J. Foster, Anja B. Frank, Qijian Li, Silvia Danise, Xia Wang, Jörn Peckmann

Corresponding author: [w.j.foster@gmx.co.uk](mailto:w.j.foster@gmx.co.uk)

**The PDF file includes:**

Materials and Methods

Supplementary Text

Figs. S1 to S13

Tables S1 to S3

Supplementary References

Supplementary Text

Materials and Methods

Fossil Data

To obtain datasets to investigate changes in fossil diversity for the Meishan sections, we retrieved species-level occurrences of marine taxa from the Geobiodiversity Database (http://www.geobiodiversity.com) and the Paleobiology Database (https://paleobiodb.org) with duplicate records removed. The database was also supplemented with references missing from both databases. To calculate the ranges of each species, we created datasets that span from the Longtan Formation (Wuchiapingian) to the Nanlinghu Formation (Dienerian). The wide breadths of these time intervals were chosen to reduce the impact of edge effects. The clades included in the dataset were the Arthropoda, Brachiopoda, Bryozoa, Chlorophyta, Chordata, Cnidaria, Foraminifera, Mollusca, Radiolaria, Rhodophyta, and Problematica.

The occurrences were manually vetted to ensure that typographic errors were corrected so species did not appear with multiple spellings, and to ensure that individual species were not represented within multiple genera in the database due to taxonomic synonymy, in which case the most up-to-date species identification of the species was followed. Freshwater and palynomorph fossils were removed.

To calculate the stratigraphic range of each species, occurrences of species with open nomenclature (“”, ?, aff., cf., informal) were included. Occurrences designated as indeterminate species (e.g., sp. or spp.) were included and assumed to represent a separate species. Occurrences that were only identified to the genus-level or higher were excluded. The latter was done to avoid taxonomic artefacts in the reassignment of species: when a species is reassigned to a new genus, its occurrences are moved to that genus, whereas a genus-level assignment is left in the original genus. In older references from the Meishan section the beds do not have the same stratigraphic subdivision as today, and the occurrence of a specimen is considered present in all respective sub-beds. These occurrences that were not specified to a single bed/ sub-bed as recognized in this study were subsequently excluded as the timing of extinction is poorly constrained.

The resulting database included 603 species from 6457 occurrences.

Inorganic Geochemical Data

To obtain datasets to investigate changes in environmental conditions, we downloaded the raw datasets of inorganic geochemical proxies for the Meishan sections. This was done by a manual literature search.

We initially obtained all the articles for each proxy investigated for the Meishan sections. Where multiple records of a single proxy were collected, we avoided mixing datasets collected from the same beds by different studies and selected the most extensive record. For example, multiple records of carbon isotopes exist for the Meishan section, but we used the record developed primarily by Cao Changqun, which provides the most complete carbon isotope record for the Meishan sections. This was done to avoid differences between studies being analytical artefacts from analyses run on different instruments. In cases where the raw datasets were not published/ publicly accessible, we either contacted the corresponding author or used a subsequent study that published the data. For example δ^13^C_carb_  from Cao et al. (2009) is not publicly available but the data is subsequently published in Shen et al. (2013). The way the geochemical data is reported from the Meishan sections also varies between studies. Some only report the log height, some only the bed number, and other report all of the stratigraphical information. For studies that only report the bed number (e.g., Song et al., 2012) we use the mid-height of the corresponding bed number. The resulting database included 18 proxies (Table S1).

The samples heights were standardized according to the Permian/Triassic boundary with 0 cm marking the base of bed 27c. Data from the Meishan core, which is located 550 m west of the Meishan section D (GSSP section), were scaled to correlate with the section from Meishan D as the beds demonstrate considerable thickness variations.

**Table S1: The different proxies that were evaluated in this study and the source of the data that was used in the analysis.** The interpretations of each proxy follow the original reference, but note that no geochemical proxy unambiguously reflects a single environmental variable.

| **Proxy** | **Environmental change** | **Interpretation** | **Reference** |
| --- | --- | --- | --- |
| δ^7^Li | Weathering | Influx of isotopically light Li (negative excursion) at Meishan corresponds with a fluvial influx and indicates an enhanced weathering rate. | Sun et al., 2018 |
| δ^13^C_carb_ | Volcanic carbon | Influx of isotopically light C at Meishan interpreted as a carbon injection from the Siberian Traps. | Shen S-Z et al., 2013 |
| δ^13^C_org_ | Volcanic carbon |  | Cao et al., 2009 supplemented at beds 23-38 with Huang et al., 2007 and Sial et al., 2021. |
| δ^15^N | Primary productivity | Influx of light N at Meishan are hypothesised to signal significantly enhanced cyanobacterial N-fixation. | Cao et al., 2009 |
| δ^18^O_apatite_ | SST temperature | Shift to negative values indicates an increase in sea surface temperatures. | Chen et al., 2016 |
| δ^34^S | Redox conditions | Negative δ^34^S and positive Δ^33^S values reflect lower degrees of sulphate reduction in open-system environments | Shen Y et al., 2011 |
| ∆^33^S | Redox conditions |  | Shen Y et al., 2011 |
| δ^44/40^Ca_apatite_ | Ocean acidification | Negative shift in the δ^44/40^Ca interpreted as a shift in the δ^44/40^Ca of seawater and development of ocean acidification. | Hinojosa et al., 2012 |
| δ^66^Zn_carb_ | Volcanic Zn | Abrupt negative δ^66^Zn shift are a consequence of a rapid increase of Zn from ^66^Zn-depleted external sources (volcanic ashes, and/or extremely fast weathering of fresh LIPs.). | Liu et al., 2017 |
| ^87^Sr/^86^Sr_apatite_ | Weathering | Rise of ^87^Sr/^86^Sr ratios reflect a rapid increase of riverine influx of Sr caused by intensified weathering. | Song et al., 2015 |
| δ^114/110^Cd | Primary productivity | Negative shift interpreted as a decline in nutrient utilization by phytoplankton and a reduction in primary productivity. | Zhang et al., 2018 |
| ^187^Os/^188^Os | Volcanic Os | Increased input of unradiogenic Os from volcanism. | Liu et al., 2020 |
| TOC | Unspecific | TOC is related to many factors, but increased TOC has been related to increased organic carbon burial as a consequence of anoxia. | Beds 4a to 22 Cao et al., 2009 and Beds 22 to 34 from Zhang et al., 2018. |
| Hg/TOC | Volcanic Hg | The source of organic bound Hg was attributed to the Siberian Traps and increased values indicate volcanic episodes. | Sial et al., 2021 |
| Th/U_apatite_ | Redox conditions | Increased values indicate development of anoxic conditions. | Song et al., 2012 |
| ΩCe_apatite_ | Redox conditions | Values below -0.1 used to indicate oxic to dysoxic conditions. | Song et al., 2012 |
| Fe_HR_/Fe_total_ | Redox conditions | Values >0.38 are used to indicate anoxic conditions | Xiang et al., 2020 |
| Fe_pyrite_/Fe_HR_ | Redox conditions | Fe_HR_/Fe_T_ >0.38 values and Fe_py_/Fe_HR_ >0.7 is used to indicate a euxinic water column, whilst values <0.7 indicate ferruginous conditions. | Xiang et al., 2020 |

Estimating the number of extinction pulses

To quantify the nature of extinction pulses, here we used a modified version of the two-step extinction pulse algorithm of Wang and Zhong (2018). Prior to analysis, singletons and doubletons were removed from the analysis, because their confidence intervals cannot be calculated. The number of extinction pulses was initially calculated separately for Brachiopoda, Mollusca, Arthropoda, Foraminifera and Conodonta. Subsequently, the total dataset was divided into 10 splits with 70% of the whole dataset randomly selected. This was done for two reasons, (1) the large dataset of fossils from Meishan is computationally expensive and requires unfeasible running times, and (2) subdividing the main dataset and running the experiments over 10 splits allows us to investigate how changes in the database completeness affects our interpretations.

These datasets are still large with long computationally running times and require a large amount of memory to store the partitions generated by the data. Therefore, the algorithm from Wang and Zhong (2018) was modified to be more efficient by the use of parallel computation using the mclapply() function. Note, the mclapply() function only works with macOS and Unix operating systems (i.e., not on Windows). We did not use the parLapply() function that works for Windows because mclapply() generally outperforms parLapply(). The mclapply() function also performs better with more cores and, therefore, the Universität Hamburg computing nodes, which have 90 cores and a large memory (600 GB), were used to run the analysis and significantly reduced the running time (1 hour 10 minutes per split).

The number of maximum pulses (*k*) is set to affects the results and also the running time duration. For example, with 233 taxa, there is: 1 way to partition 233 taxa among 1 pulse; 232 ways to partition 233 taxa among 2 pulses; 26796 partitions among 3 pulses; 2,054,360 partitions among 4 pulses etc. Therefore, rather than setting *k* at an arbitrary high value, *k* was set as 1 more than the number of pulses determined by the confidence intervals for the analysis between different taxonomic groups, and *k* was set at 4 when investigating the splits of the total dataset. The number of estimated pulses is also affected by background extinctions prior to the mass extinction (Wang and Zhong 2018). Therefore, an analysis of the timing of extinction for the total dataset was only investigated where taxa that went extinct prior to the Meishan Member were removed from the analysis. This reduced the number of the taxa in the analysis and the associated running times, and allowed the algorithm to focus on the number of extinction pulses around the Permian/Triassic boundary.

**Table S2: Results for the 10 splits looking at the timing of extinction at the Meishan section.** Confidence levels for each split are shown and the bed number(s) is also given when confidence levels are above 0.00.

|  | **0 pulses (*k* = 1)** | **1 pulse (*k* = 2)** | **2 pulses (*k* = 3)** | **3 pulses (*k* = 4)** |
| --- | --- | --- | --- | --- |
| Split 1 | 0.00 | 0.65 (bed 29a) | 0.25 (+ bed 23a) | 0.10 (+ bed 27d) |
| Split 2 | 0.00 | 0.90 (bed 29a) | 0.10 (+ bed 23a) | 0.00 |
| Split 3 | 0.00 | 0.75 (bed 29a) | 0.25 (+ bed 23a) | 0.00 |
| Split 4 | 0.00 | 0.65 (bed 29a) | 0.25 (+ bed 23a) | 0.10 (+ bed 27d) |
| Split 5 | 0.00 | 0.70 (bed 29a) | 0.30 (+ bed 23a) | 0.00 |
| Split 6 | 0.00 | 0.75 (bed 29a) | 0.20 (+ bed 23a) | 0.05 (+ bed 21) |
| Split 7 | 0.00 | 0.75 (bed 29a) | 0.20 (+ bed 23a) | 0.05 (+ bed 28) |
| Split 8 | 0.00 | 0.75 (bed 29a) | 0.25 (+ bed 23a) | 0.00 |
| Split 9 | 0.00 | 0.65 (bed 29a) | 0.30 (+ bed 23a) | 0.05 (+ bed 21) |
| Split 10 | 0.00 | 0.65 (bed 29a) | 0.35 (+ bed 23a) | 0.00 |

Estimating changes in species richness

The species richness diversity curve was based on boundary-cross diversity (N_bL_+ N_Ft_ + N_bt_, where N_bL_ is the number that cross the bottom boundary only, N_Ft_ is the number that cross the top boundary only, and N_bt_ is the number that cross both boundaries.), i.e., species richness diversity curve excluded species that only occur in one time interval. Extinction rate follows the per capita extinction rates of Foote (2001) (−log[N_bt_/(N_bL_ + N_bt_)]).

Segmented regression analysis

The data included in the segmented data analysis was every available data point from Bed 1 (-4390 cm) to bed 34 (+773 cm). To quantitatively determine the number of breakpoints in the segmented regression analysis, the selgmented() from the segmented package in R was used (Muggeo et al., 2014). The segmented() function was then used to determine where these breakpoints occur for each geochemical proxy. The raw data and segmented regressions were then plotted using ggplot2 package in R.

Data Imputation

We compared the results of data imputation between 2-point interpolations (where a value is interpolated based on the value immediately above and below the missing value), segmented regression models (where the missing value is based on the linear regression model), and predictive means matching (where missing values are estimated based on the relationship of the data between all complete cases of the other proxies). Each imputation method has different underlying assumptions and produce variable results. Figs. S9-S10 show a comparison of the raw data with the different data imputation methods. Predictive means matching appears to give the most variable missing value estimates that vary the most from the raw data, whereas 2-point interpolations and segmented regressions give similar results for proxies with high-resolution datasets. For proxies with poor sampling, e.g. δ^15^N, all the methods give variable results.

The segmented regression data imputation method was used in computing missing values because (a) it is less affected by anomalous datapoints, (b) estimates are based upon overall trends in the data, (c) it does not assume that a relationship between different proxies exists, and (d) it recognizes significant shifts in data trends and is more dynamic than a single regression model.

We also explored the data for significant correlations between the different proxies (Fig. S11-13). Comparison of these plots between the average values for each bed and the imputed missing values shows that the imputed values slightly reduced the correlation between the proxies, but the overall patterns remain the same. When only the beds 21-29a were included, i.e., the beds used in our subsequent analysis, far fewer significant correlations exist within the data (Fig. S13).

Multiple linear regression models

Before developing the multiple linear model it is first essential to investigate which proxies should be included in the model, i.e., which proxies correlate with changes in diversity and per capita extinction rates. To investigate the impact of the imputed values and the selection of which beds to include in the analysis, comparisons were made when comparing changing in species richness and proxies (Tab. S3). The proxies that had significant correlations at *p* < 0.001 were not affected by the imputed values nor reducing the number of beds included in the analysis. Reducing the beds that were included in the analysis to beds 22-29a created more proxies with significant correlations at *p* < 0.05. The imputed dataset only had a drastic change with the significance of the Th/U_apatite_ proxy, which can likely be attributed to the large amount of imputed values.

**Table S3: Individual regression coefficients and *p* values for each geochemical proxy and the diversity metrics.** Significant relationships are highlighted in gray. Species richness was calculated against mean proxy values for each bed, whereas per-capita extinction rates were compared with the variance of each geochemical proxy.

| Proxy | Raw Data Species Richness (beds 6-29a) | | Raw Data Species Richness (beds 22-29a) | | Imputed Species Richness (beds 22-29a) | | Per-capita extinction rate (beds 22-29a) | |
| --- | --- | --- | --- | --- | --- | --- | --- | --- |
| δ^13^C_carb_ | 0.62 | < 0.001 | 0.61 | < 0.001 | 0.62 | < 0.001 | 0.07 | 0.320 |
| δ^13^C_org_ | 0.01 | 0.642 | 0.20 | 0.084 | 0.20 | 0.084 | 0.00 | 0.974 |
| δ^18^O_apatite_ | 0.72 | < 0.001 | 0.71 | < 0.001 | 0.71 | < 0.001 | 0.25 | 0.562 |
| ∆^33^S | 0.02 | 0.650 | 0.02 | 0.650 | 0.04 | 0.472 | 0.04 | 0.455 |
| δ^34^S | 0.16 | 0.170 | 0.16 | 0.170 | 0.19 | 0.094 | 0.08 | 0.314 |
| δ^7^Li | 0.11 | 0.342 | 0.11 | 0.342 | 0.18 | 0.099 | 0.01 | 0.698 |
| δ^15^N | 0.12 | 0.165 | 0.53 | 0.041 | 0.69 | < 0.001 | 0.05 | 0.402 |
| δ^44-40^Ca | 0.55 | 0.002 | 0.55 | 0.002 | 0.53 | 0.001 | 0.01 | 0.759 |
| δ^66^Zn | -0.09 | 0.997 | 0.13 | 0.334 | 0.04 | 0.462 | 0.03 | 0.543 |
| TOC | 0.21 | 0.011 | 0.02 | 0.614 | 0.02 | 0.614 | 0.18 | 0.103 |
| δ^114-110^Cd | 0.64 | < 0.001 | 0.64 | < 0.001 | 0.64 | < 0.001 | 0.28 | 0.043 |
| ^87^Sr/^86^Sr_apatite_ | 0.05 | 0.360 | 0.01 | 0.822 | 0.00 | 0.856 | 0.00 | 0.809 |
| ^187^Os/^188^Os | 0.27 | 0.069 | 0.19 | 0.176 | 0.18 | 0.102 | 0.02 | 0.587 |
| Hg/TOC | 0.22 | 0.208 | 0.22 | 0.208 | 0.27 | 0.121 | 0.36 | 0.090 |
| Fe_HR_/Fe_tot_ | 0.36 | 0.019 | 0.32 | 0.036 | 0.33 | 0.019 | 0.02 | 0.602 |
| Fe_Py_/Fe_HR_ | 0.26 | 0.051 | 0.29 | 0.049 | 0.29 | 0.030 | 0.03 | 0.517 |
| Th/U_apatite_ | 0.26 | 0.012 | 0.06 | 0.609 | 0.29 | 0.032 | 0.01 | 0.723 |
| ΩCe_apatite_ | 0.01 | 0.732 | 0.59 | 0.044 | 0.33 | 0.020 | 0.02 | 0.623 |


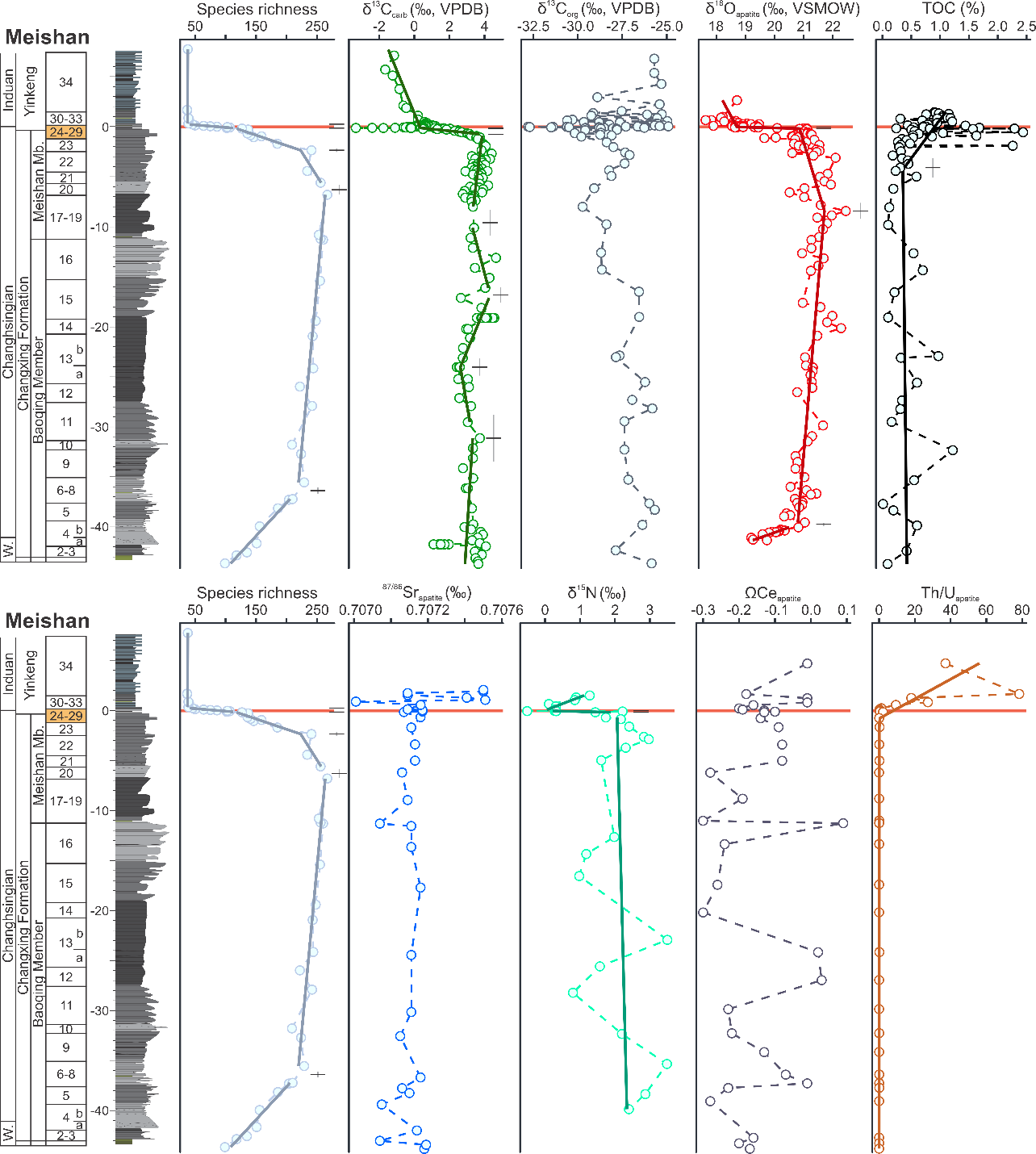


**Fig. S1.** **Stratigraphic correlation of paleoenvironmental proxies with species diversity at the Meishan sections, South China, with segmented regression lines overlain.** Breakpoints and standard errors are shown next to the regression segments. See Table S1 for references of the different proxies.


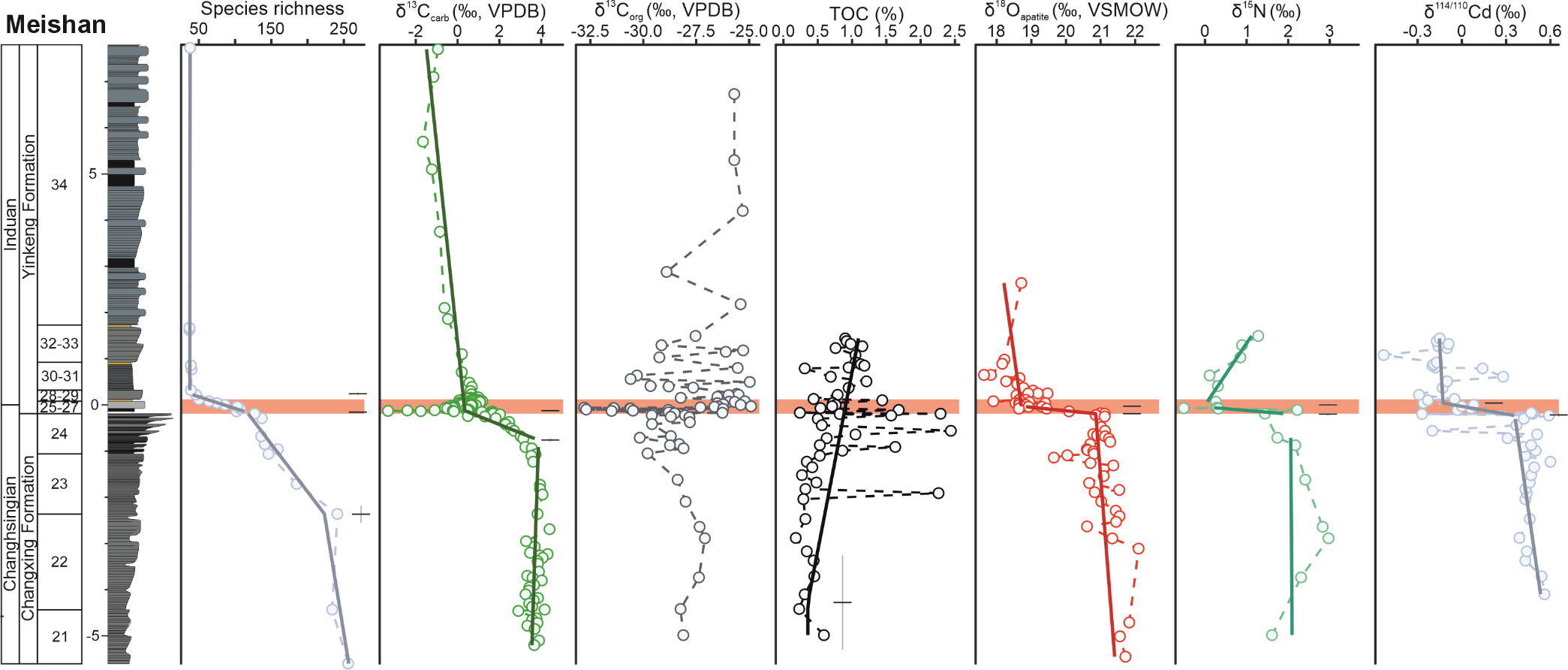


**Fig. S2.** **Stratigraphic correlation of paleoenvironmental proxies with species diversity at the Meishan sections, South China, with segmented regression lines overlain.** Breakpoints and standard errors are shown next to the regression segments. See Table S1 for references of the different proxies.


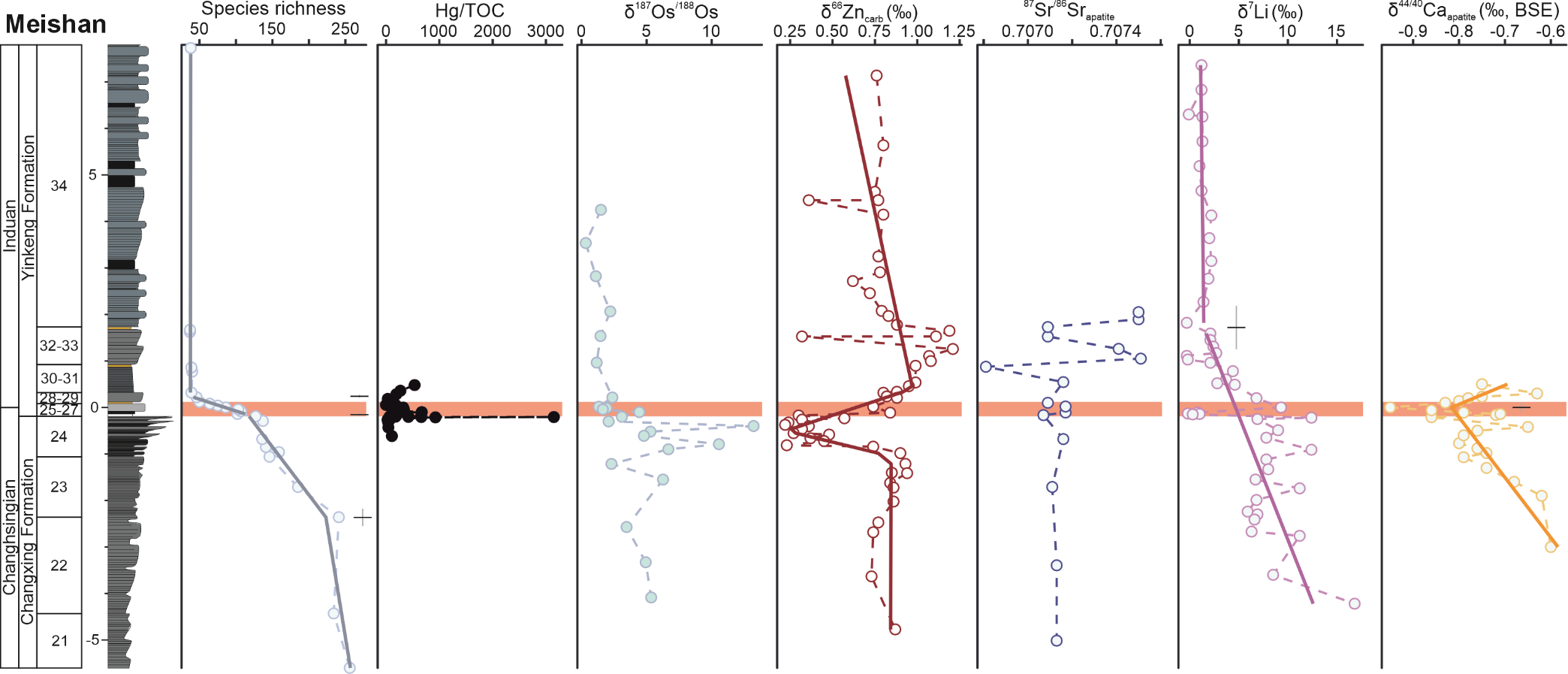


**Fig. S3.** **Stratigraphic correlation of paleoenvironmental proxies with species diversity at the Meishan sections, South China, with segmented regression lines overlain.** Breakpoints and standard errors are shown next to the regression segments. See Table S1 for references of the different proxies.


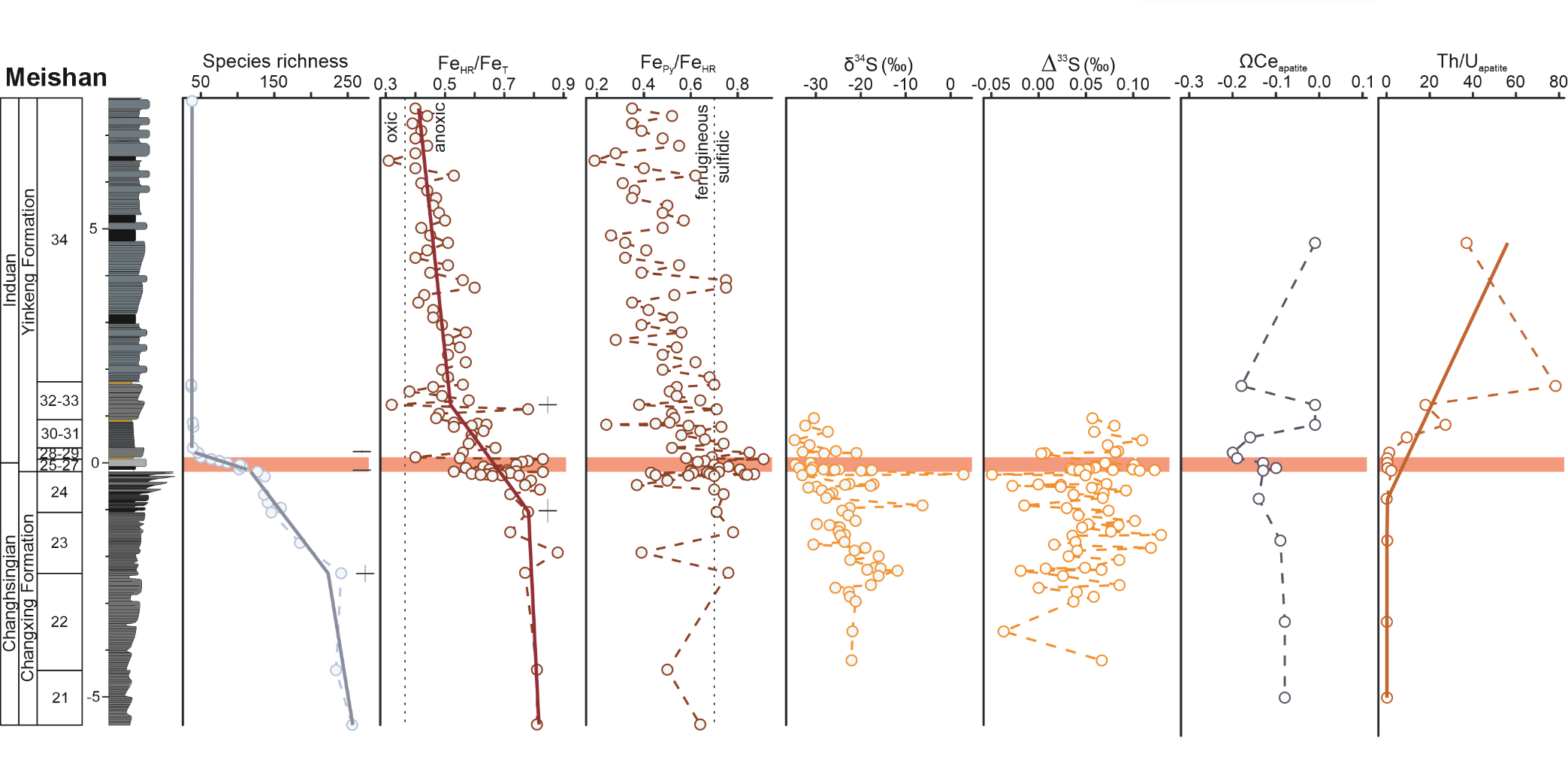


**Fig. S4.** **Stratigraphic correlation of paleoenvironmental proxies with species diversity at the Meishan sections, South China, with segmented regression lines overlain.** Breakpoints and standard errors are shown next to the regression segments. See Table S1 for references of the different proxies.


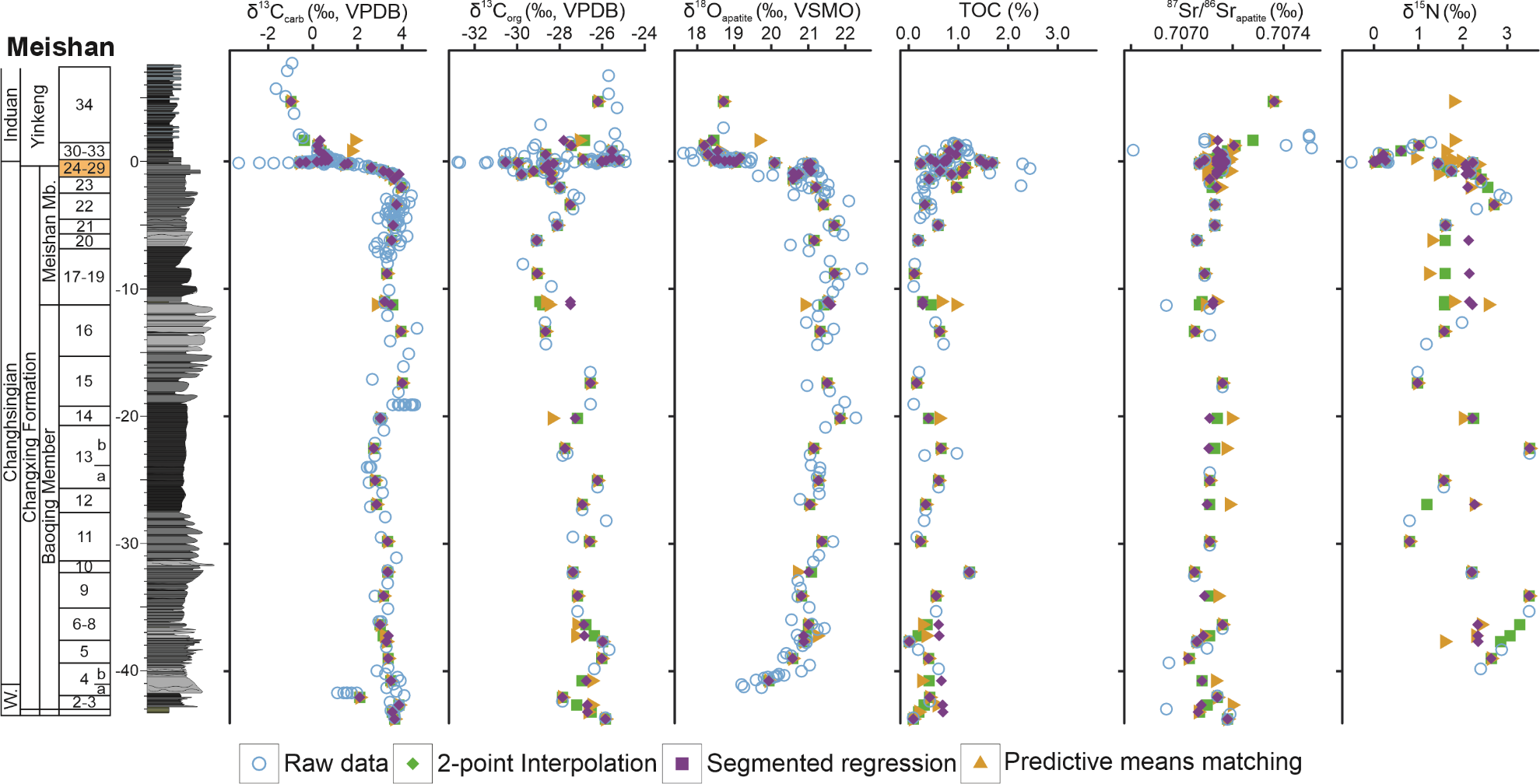


**Fig. S5.** **Data imputation comparison between the different methods for calculating values for beds missing data.** The data imputation methods are trying to create an average value for each bed. Note: when all the methods give the same result, it is because no values were calculated as that bed included raw data.


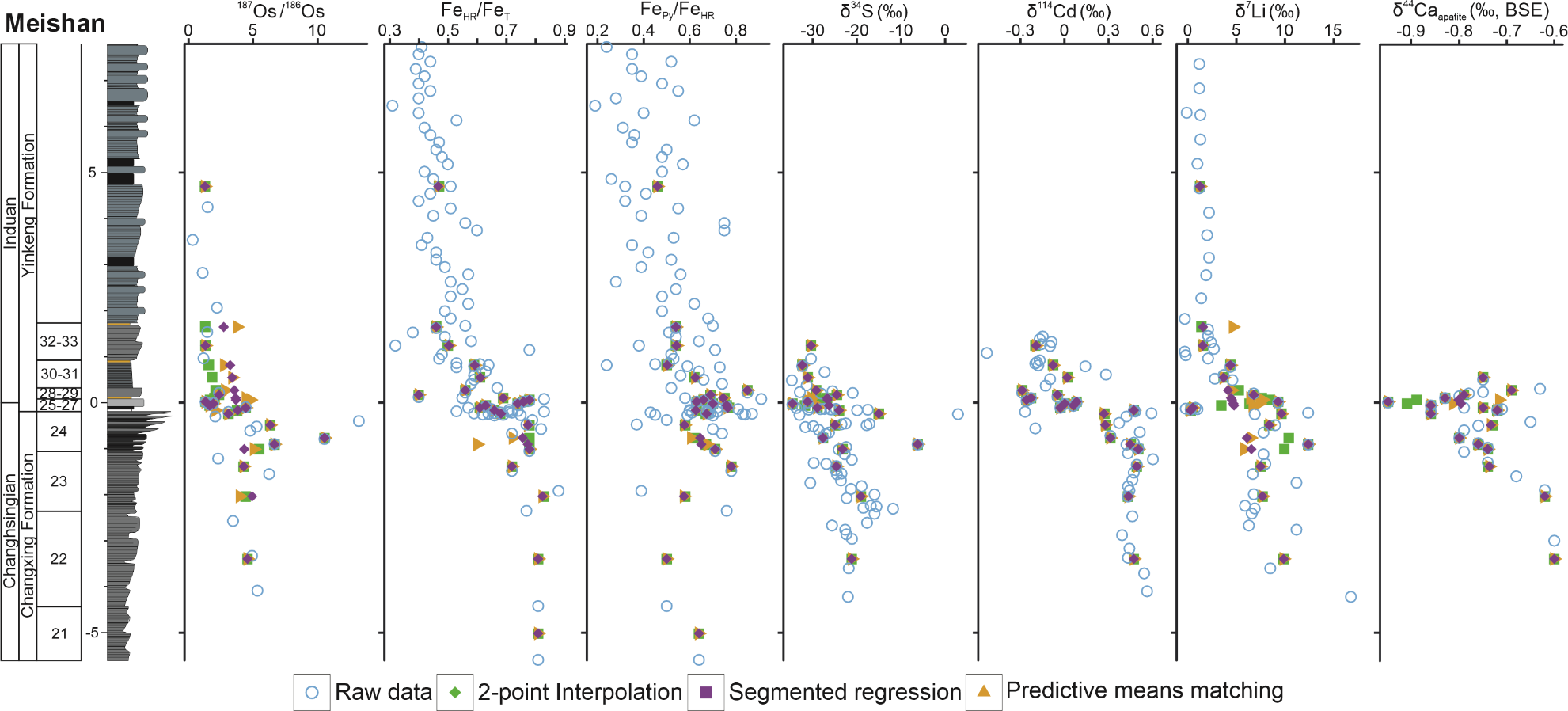


**Fig. S6.** **Data imputation comparison between the different methods for calculating values for beds missing data.** The data imputation methods are trying to create an average value for each bed. Note: when all the methods give the same result, it is because no values were calculated as that bed included raw data.


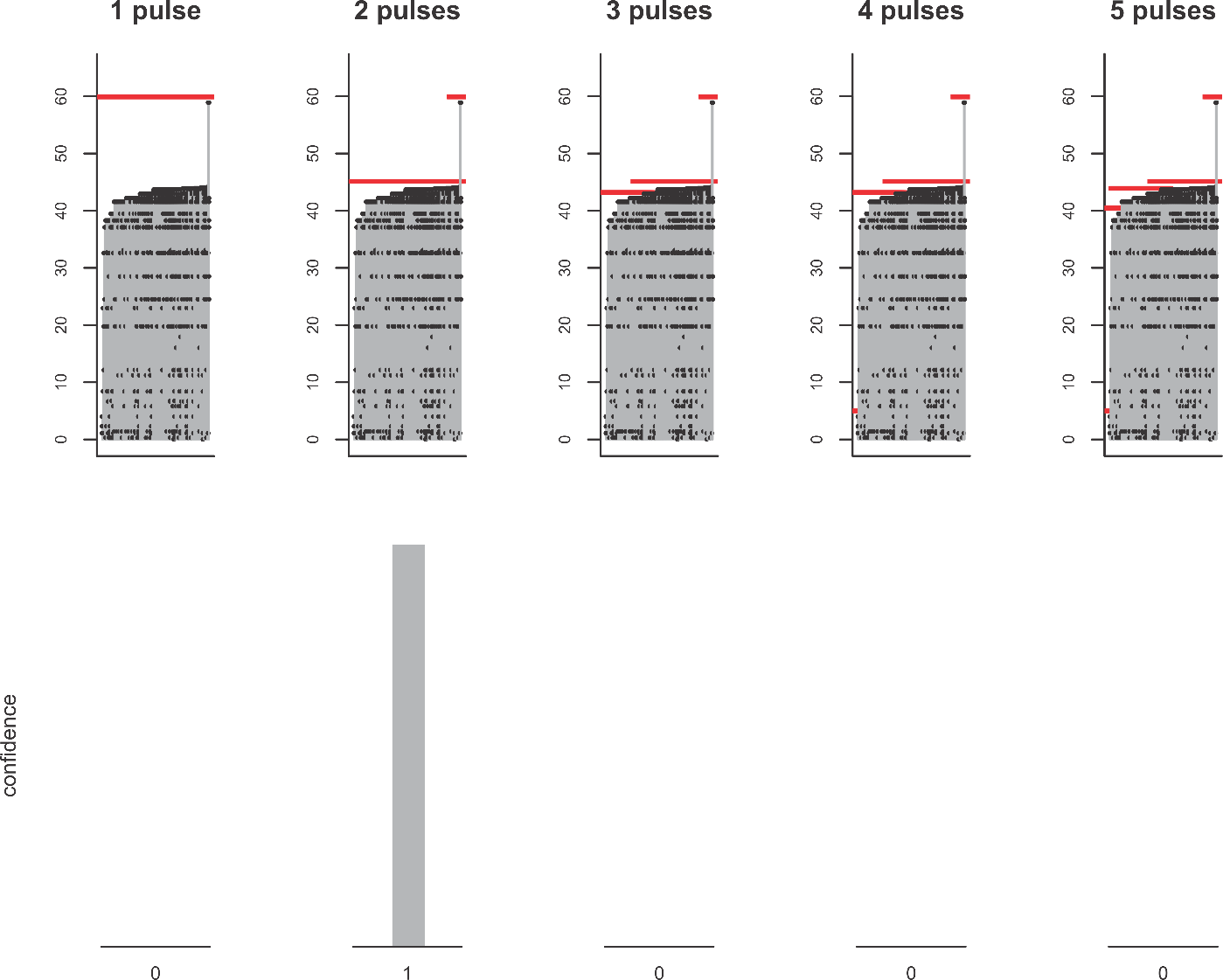


**Fig. S7.** **Range charts for Foraminifera investigating the number of extinction pulses with 125 species and 5 pulses and corresponding confidence levels (bottom row).** The y-axis is the log height of the Meishan section. Each vertical line corresponds to a taxon, with dots denoting the positions of its fossil occurrences. Red lines represent extinction pulses. The pulse at the end of the section is an edge effect and we state with 100% confidence that the extinction occurred in a single pulse at bed 29a.


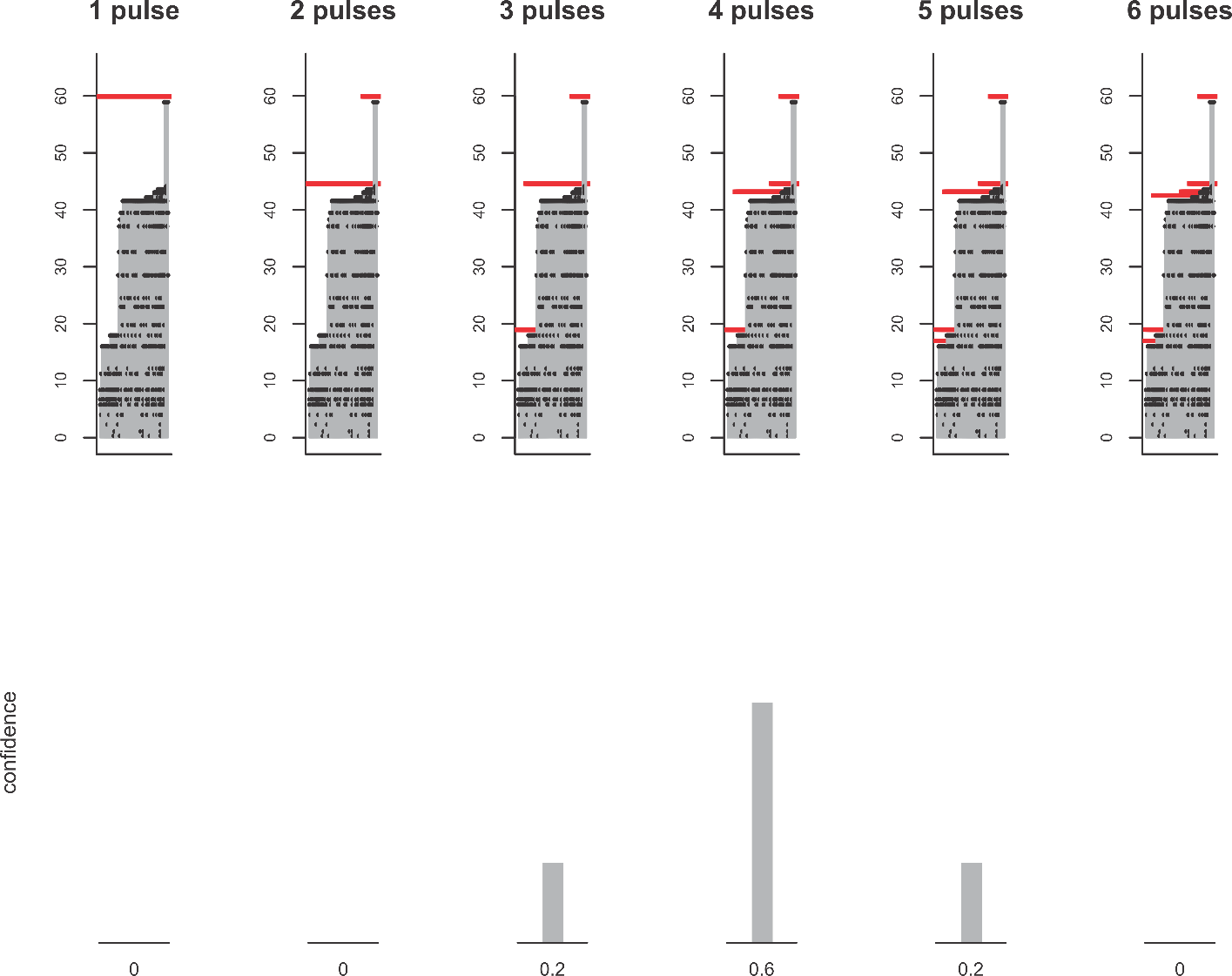


**Fig. S8.** **Range charts for Arthropoda (all ostracods except 1 trilobite species) investigating the number of extinction pulses with 76 species and 6 pulses and corresponding confidence levels (bottom row).** The y-axis is the log height of the Meishan section. Each vertical line corresponds to a taxon, with dots denoting the positions of its fossil occurrences. Red lines represent extinction pulses. The pulse at the end of the section is an edge effect and we state with 60% confidence that the extinction occurred in multiple pulses at beds 12, 23a, and 24d.


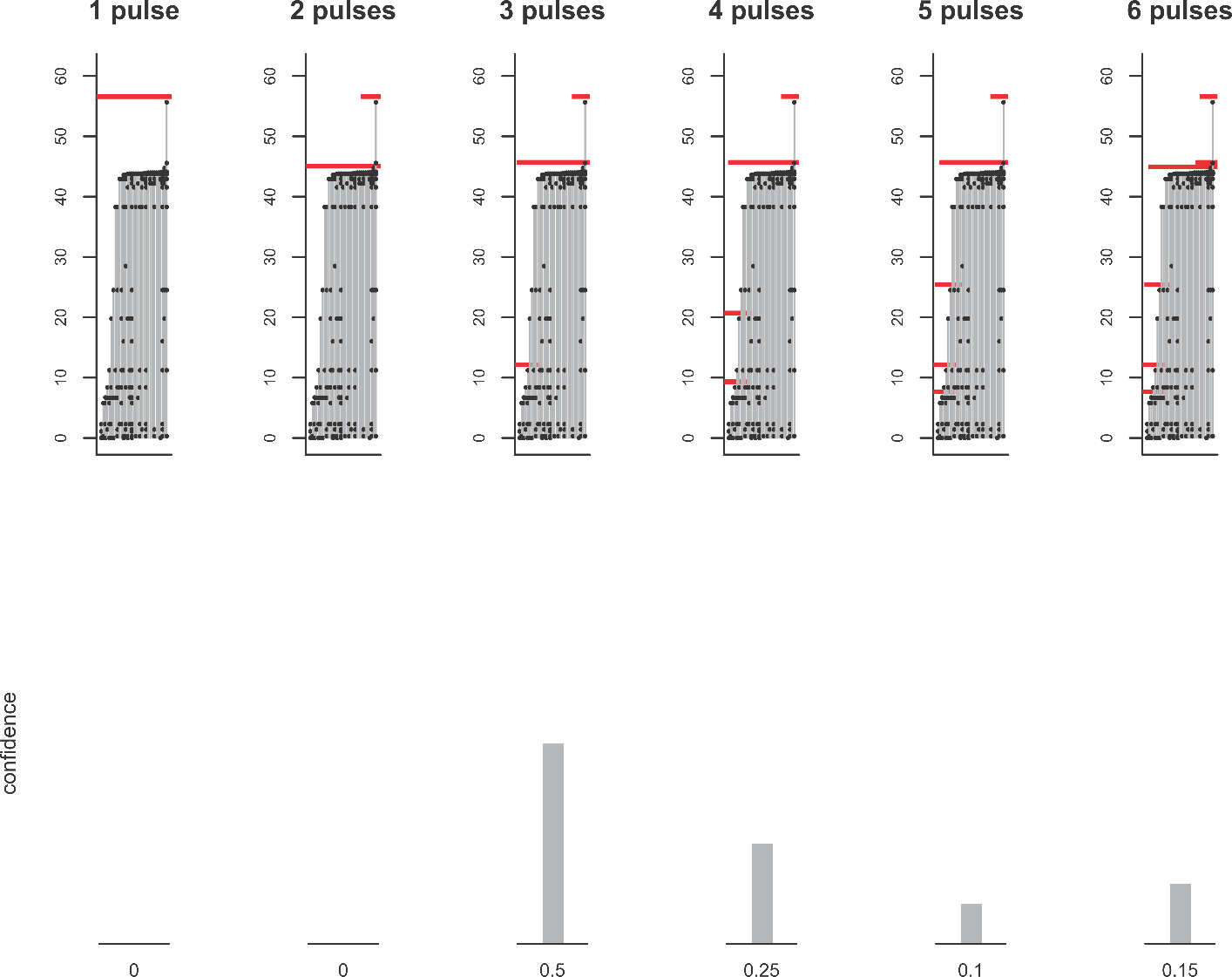


**Fig. S9.** **Range charts for Brachiopoda investigating the number of extinction pulses with 33 species and 6 pulses and corresponding confidence levels (bottom row).** The y-axis is the log height of the Meishan section. Each vertical line corresponds to a taxon, with dots denoting the positions of its fossil occurrences. Red lines represent extinction pulses. The pulse at the end of the section is an edge effect and we state with 50% confidence that the extinction occurred in multiple pulses at beds 9, and 29a.


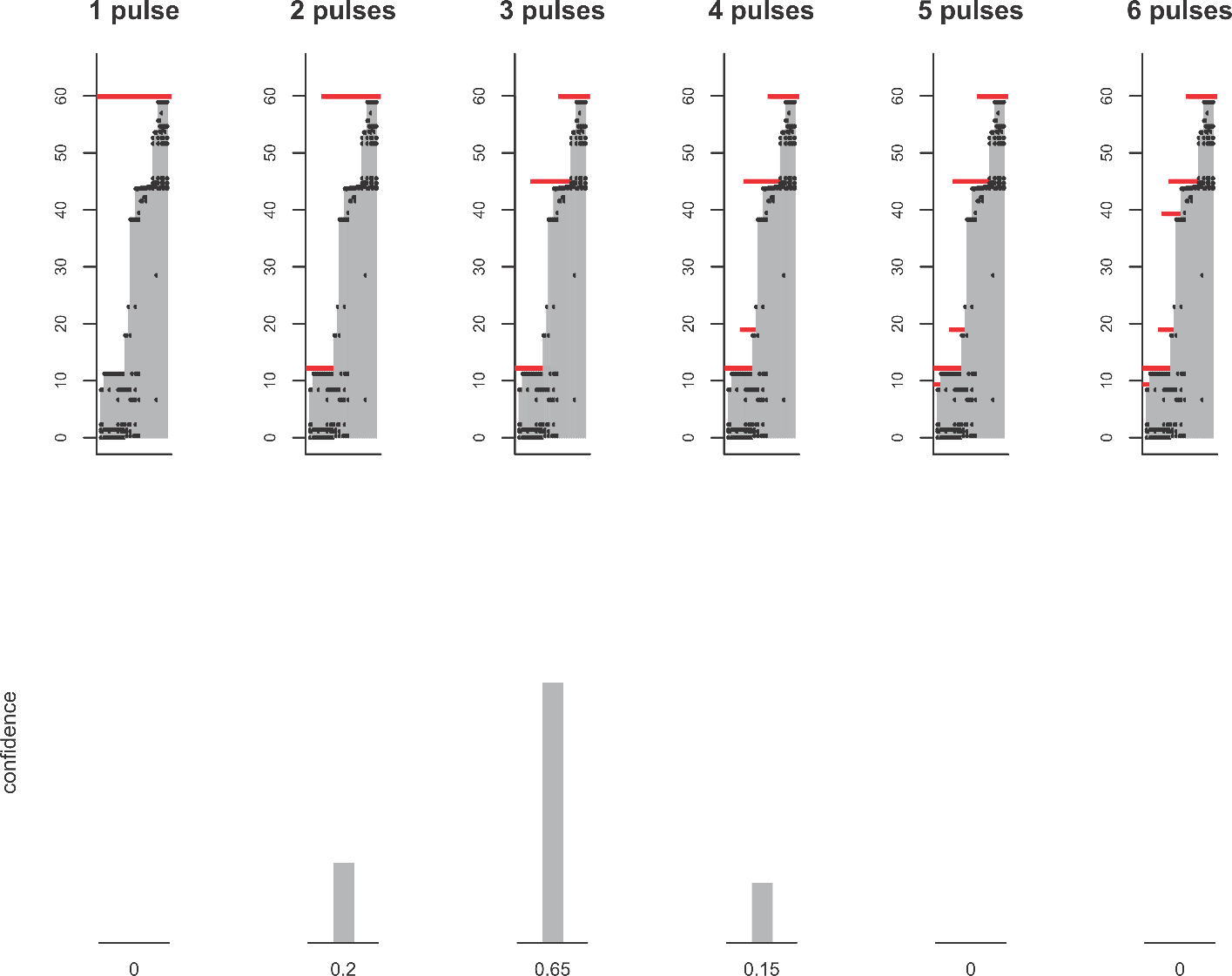


**Fig. S10.** **Range charts for Mollusca investigating the number of extinction pulses with 39 species 6 pulses and corresponding confidence levels (bottom row).** The y-axis is the log height of the Meishan section. Each vertical line corresponds to a taxon, with dots denoting the positions of its fossil occurrences. Red lines represent extinction pulses. The pulse at the end of the section is an edge effect and we state with 65% confidence that the extinction occurred in multiple pulses at beds 9 and 28.


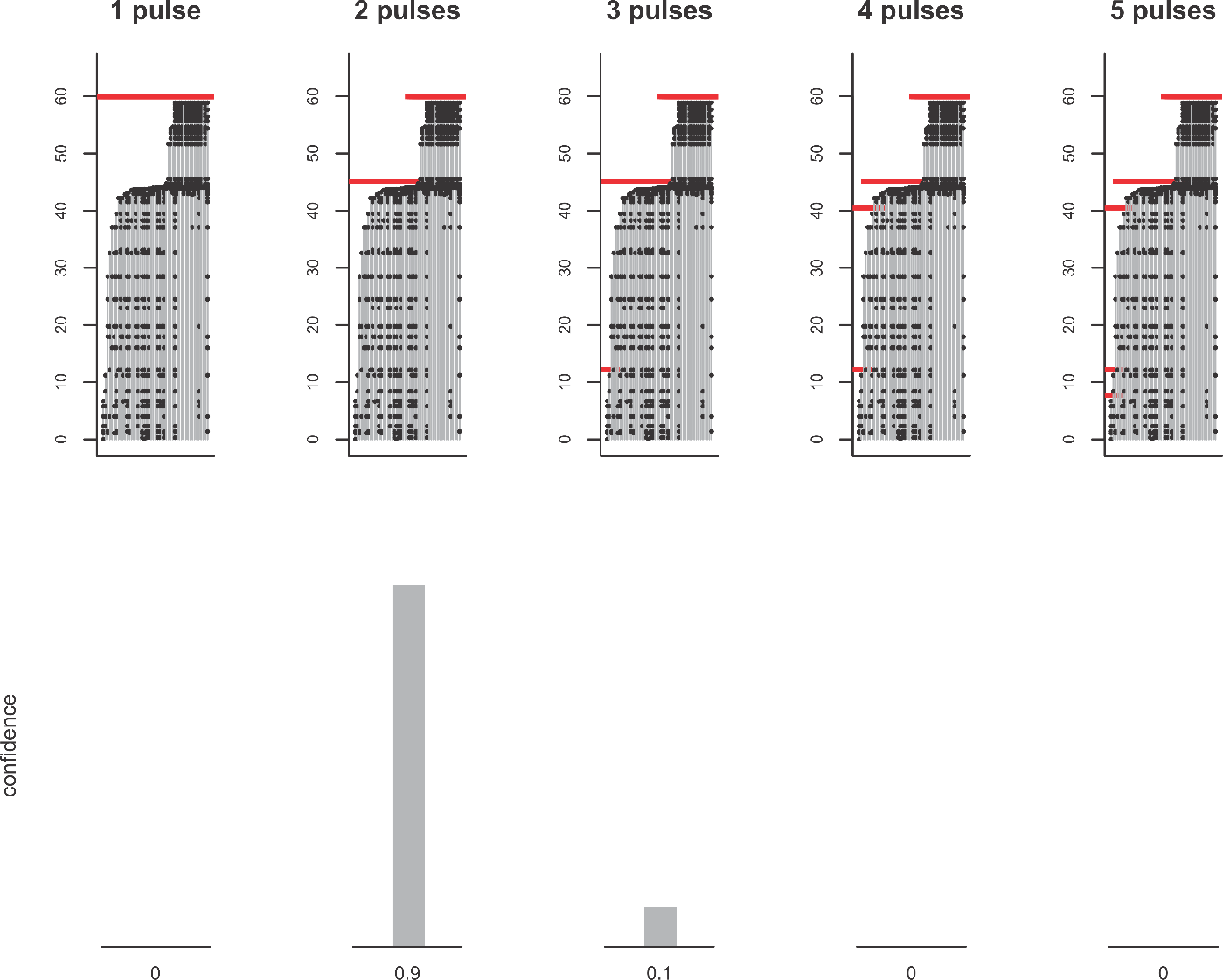


**Fig. S11.** **Range charts for Conodonta investigating the number of extinction pulses with 49 species 5 pulses and corresponding confidence levels (bottom row).** The y-axis is the log height of the Meishan section. Each vertical line corresponds to a taxon, with dots denoting the positions of its fossil occurrences. Red lines represent extinction pulses. The pulse at the end of the section is an edge effect and we state with 90% confidence that the extinction occurred in a pulse at bed 29a.


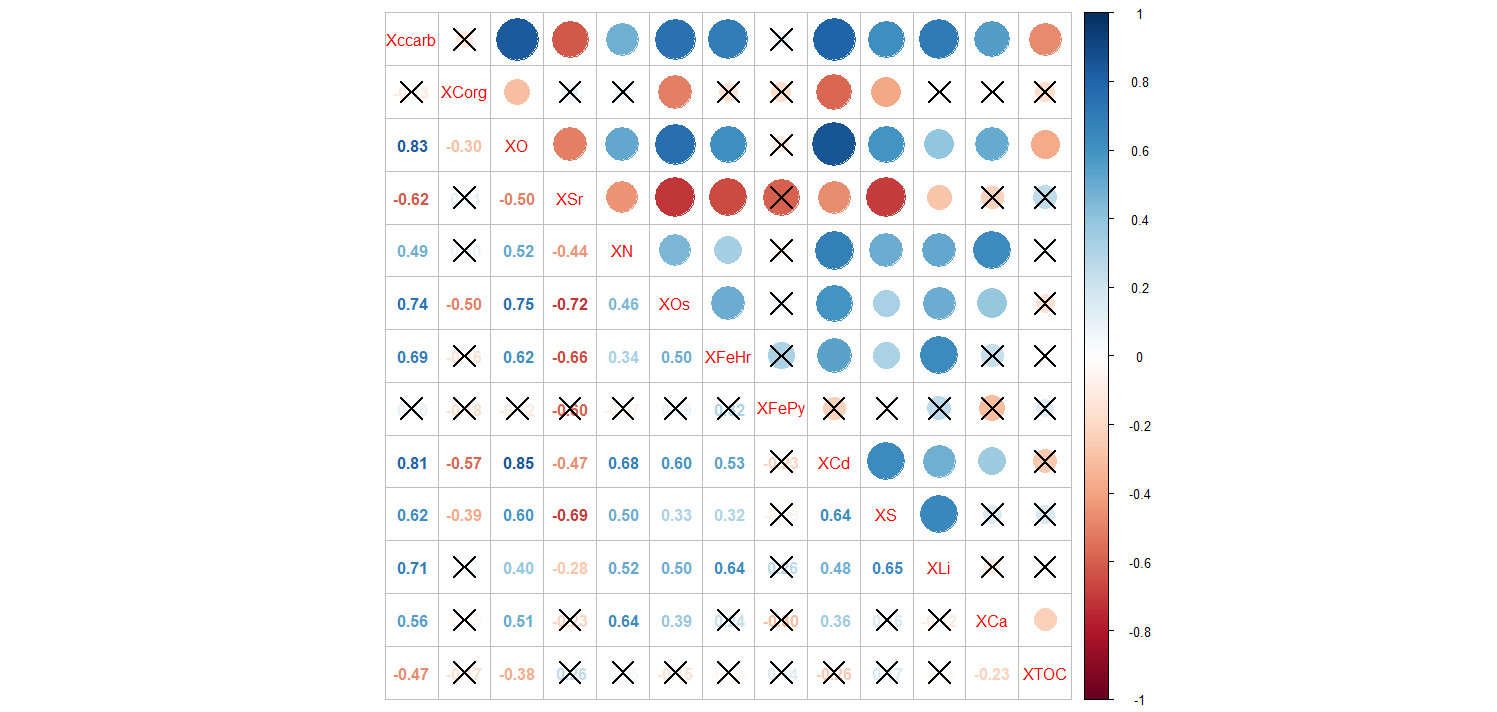


**Fig. S12.** **Multicorrelation plot of different geochemical proxies and the average values for each bed (beds with data up to bed 29a).** Correlations that are not significant are overlain with an X.


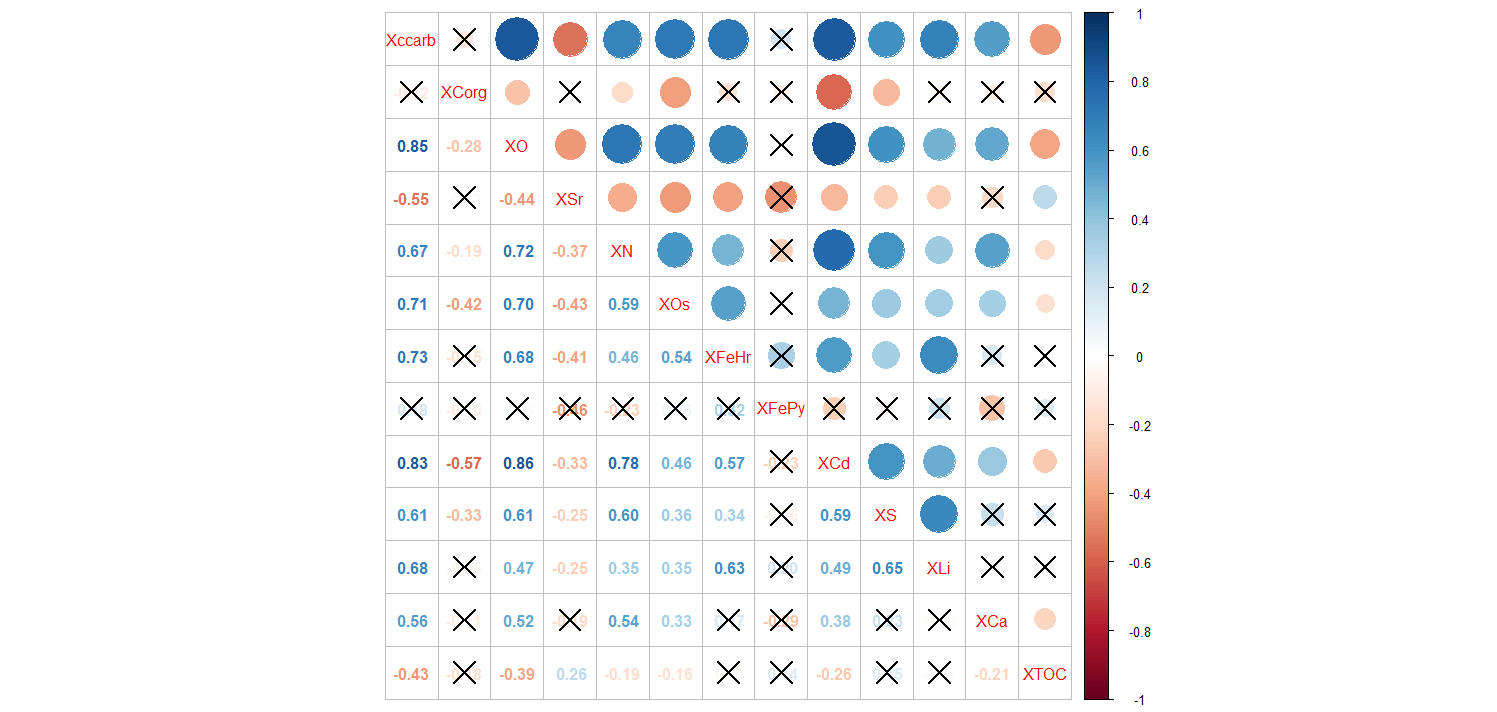


**Fig. S13.** **Multicorrelation plot of different geochemical proxies and the regression-calculated values for each bed (beds with data up to bed 29a).** Correlations that are not significant are overlain with an X.


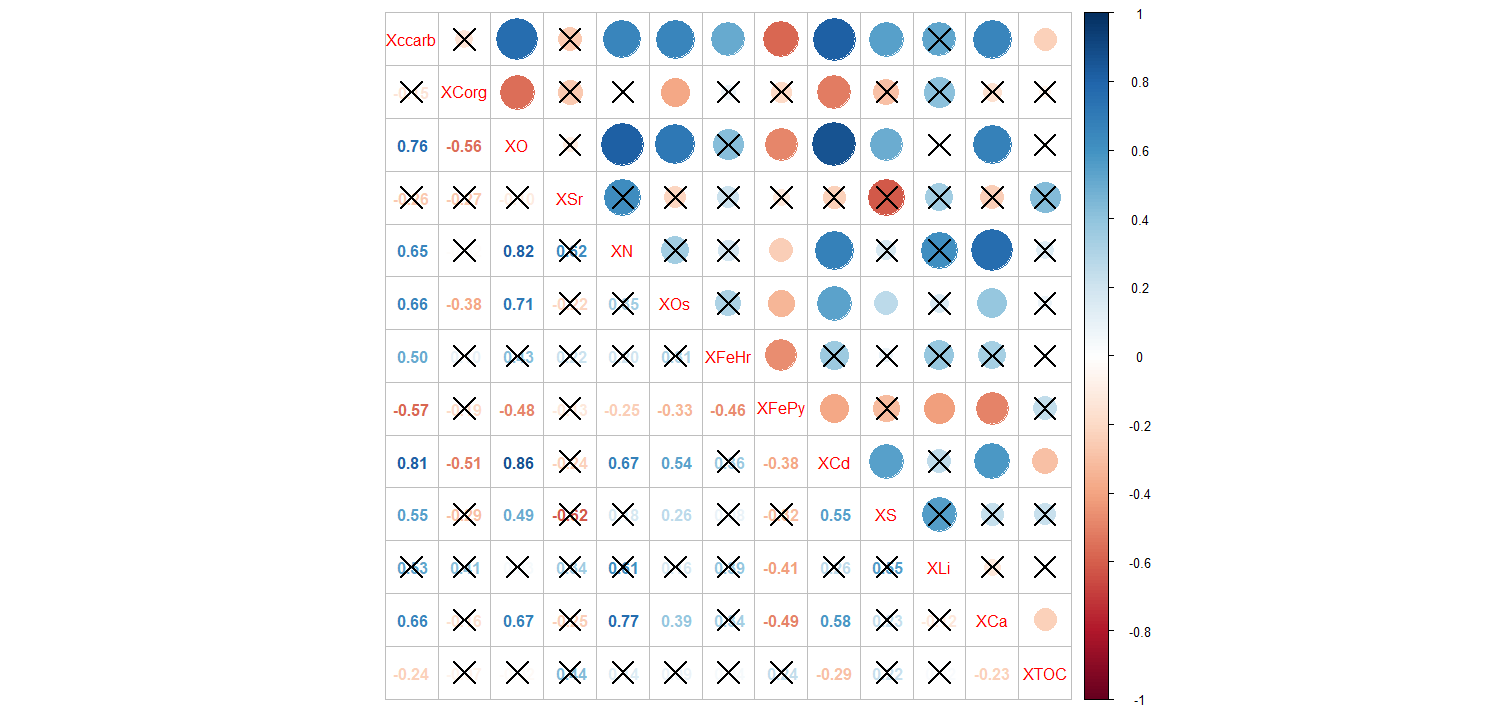


**Fig. S14.** **Multicorrelation plot of different geochemical proxies and the regression-calculated values for each bed (beds 21-29a).** Correlations that are not significant are overlain with an X.

**References**

Cao C-Q, Love GD, Hays LE, Wang W, Shen S-Z, Summons R (2009) Biogeochemical evidence for euxinic oceans and ecological disturbance presaging the end-Permian mass extinction event. Earth and Planetary Science Letters **281**, 188-201.

Chen J, Shen S-Z, Li XH, Xu YG, Joachimski MM, Bowring SA, Erwin DH, Yuan DX, Chen B, Zhang H, Wang Y, Cao C-Q, Zheng Q-F and Mu L (2016) High-resolution SIMS oxygen isotope analysis on conodont apatite from South China and implications for the end-Permian mass extinction. Palaeogeography, Palaeoclimatology, Palaeoecology **448**, 26-38.

Foote M (2001) Origination and extinction components of taxonomic diversity: general problems. Paleobiology **26**, 74-102.

Hinojosa JL, Brown ST, Chun J, DePaolo DJ, Paytan A, Shen S-Z, Payne JL (2012). Evidence for end-Permian ocean acidification from calcium isotopes in biogenic apatite. Geology **40**, 743-746.

Huang J-H, Luo G-M, Bai X, Tang X (2007) Organic fraction of the total carbon burial flux deduced from carbon isotopes across the Permo-Triassic boundary at Meishan, Zhejiang Province, China. Frontiers of Earth Science in China **1**, 425-430.

Liu S-A, Wu H, Shen S-Z, Jiang G, Zhang S, Lv Y, Zhang H, Li S (2017) Zinc isotope evidence for intensive magmatism immediately before the end-Permian mass extinction. Geology **45**, 343-346.

Liu Z-Y, Selby D, Zhang H, Shen S-Z (2020). Evidence for volcanism and weathering during the Permian-Triassic mass extinction from Meishan (South China) osmium isotope record. Palaeogeography, Palaeoclimatology, Palaeoecology **553**, 109790.

Muggeo VM, Atkins DC, Gallop RJ, Dimidjian S (2014) Segmented mixed models with random changepoints: a maximum likelihood approach with application to treatment depression study. Statistical Modelling **14**, 293-313.

Shen S-Z, Cao C-G, Zhang H, Bowring SA, Henderson CM, Payne JM, Davydov VI, Chen B, Yuan D-X, Zhang Y-C, Wang W (2013) High-resolution δ^13^C_carb_ chemostratigraphy from latest Guadalupian through earliest Triassic in South China and Iran. Earth and Planetary Science Letters **375**, 156-165.

Shen Y, Farquhar J, Zhang H, Masterson A, Zhang T, Wing BA (2011). Multiple S-isotopic evidence for episodic shoaling of anoxic water during Late Permian mass extinction. Nature Communications **2**, 210.

Sial AN, Chen J, Korte C, Pandit MK, Spangenberg JE, Silva-Tamayo JC, de Lacerda LD, Ferreira VP, Barbosa JA, Gaucher C, Pereira NS (2021) Hg isotopes and enhanced Hg concentration in the Meishan and Guryul ravine successions: Proxies for volcanism across the Permian-Triassic boundary. Frontiers in Earth Science **9**, 651224.

Song H, Wignall PB, Tong J, Bond DPG, Song H, Lai X, Zhang K, Wang H, Chen Y (2012) Geochemical evidence from bio-apatite for multiple oceanic anoxic events during Permian–Triassic transition and the link with end-Permian extinction and recovery. Earth and Planetary Science Letters **353–354**, 12–21.

Song H, Wignall PB, Tong J, Song H, Chen J, Chu D, Tian L. (2015) Integrated Sr isotope variations and global environmental changes through the Late Permian to early Late Triassic. Earth and Planetary Science Letters **424**, 140-147.

Sun H, Xiao Y-L, Gao Y-J, Zhang G-J, Casey JF, Shen Y-Y (2018) Rapid enhancement of chemical weathering recorded by extremely light seawater lithium isotopes at the Permian–Triassic boundary. Proceedings of the National Academy of Sciences **115**, 3782-3787.

Wang SC and Zhong L (2018) Estimating the number of pulses in a mass extinction. Paleobiology **44**, 199-218.

Xiang L, Zhang H, Schoepfer SD, Cao C-G, Zheng Q-F, Yuan D-X, Cai Y-F, Shen S-Z (2020). Oceanic redox evolution around the end-Permian mass extinction at Meishan, South China. Palaeogeography, Palaeoclimatology, Palaeoecology **544**, 109626.

Zhang Y, Wen H, Zhu C, Fan H, Cloquet C (2018) Cadmium isotopic evidence for the evolution of marine primary productivity and the biological extinction event during the Permian-Triassic crisis from the Meishan section, South China. Chemical Geology **481**, 110-188.
